# Interobserver Variability in Semen Analysis: Findings From a Quality Control Initiative

**DOI:** 10.7759/cureus.46388

**Published:** 2023-10-02

**Authors:** Kumar Siddharth, Tribhuwan Kumar, Md. Zabihullah

**Affiliations:** 1 Physiology, All India Institute of Medical Sciences (AIIMS) Patna, Patna, IND

**Keywords:** male infertility, andrology, interobserver variability, internal quality control, semen analysis

## Abstract

Introduction

For laboratory tests, precision and accuracy are indispensable to ensure reliable results for both clinical diagnosis and research endeavors. The accuracy and reliability of results are important because they have an impact on both patient management and research. In this study, we evaluated the inter-observer variability between a trained technician and two academic residents, which acted both as a quality control measure as well as an assessment of training outcomes.

Material and methods

Freshly obtained semen samples from 28 subjects coming to the andrology laboratory were used. Semen analysis was performed by a regular technician permanently posted in the laboratory and two residents on completion of their posting in the andrology laboratory. All three examined the same sample after liquefaction for assessment of sperm motility, sperm concentration, sperm vitality, and sperm morphology. Semen analysis was done as per the recommendations of the WHO.

Results

The results of the study are presented as a coefficient of variation (CV), S charts, and Bland-Altman plot where we evaluated the interobserver variability in parameters on semen analysis of the same sample by three different assessors. The mean CV for sperm concentration across the samples was 6.24%. For sperm vitality, sperm morphology, and sperm motility the mean CV was 10.14%, 2.66%, and 8.11%, respectively. The S chart and Bland-Altman plot found a few random errors in measurements.

Conclusion

Regular quality control assessments are essential and should be implemented in andrology laboratories to ensure accurate and reliable results. Proper training of laboratory personnel is also vital for consistent outcomes. Other measures such as equipment calibration, use of high-quality reagents, and standard reporting are also crucial for the best results from a laboratory.

## Introduction

In the field of laboratory sciences, precision and accuracy are indispensable to ensure reliable results for both clinical diagnosis and research endeavors. Internal Quality Control (IQC) is a central framework within laboratories, serving as a systematic approach to monitor and maintain the accuracy and reliability of analytical processes. The implementation of robust IQC protocols not only enhances laboratory credibility but also safeguards patient care. IQC in andrology laboratories is particularly important because of the very complex nature of semen analysis, the diverse nature of male reproductive parameters, and the subjectivity involved [1,2]. The accuracy and reliability of results are important because they influence clinical decisions, patient management, and research outcomes. The WHO Laboratory Manual for the Examination and Processing of Human Semen provides comprehensive guidelines for the analysis and handling of human semen samples in clinical and research settings. The manual underlines the critical role of quality control measures in ensuring the accuracy of semen analysis results [3,4].

There are several statistical methods for analyzing systematic errors within and between technicians. The creation of control charts and other statistical tests such as the X-bar chart, S chart, Bland-Altman plot, Youden plots, coefficient of variation (CV), and analysis of variance (ANOVA) is important for detecting an overall variation of results between technicians [2,4,5].

Our educational institution offers a diverse range of degree programs across multiple departments. The enrolled academic residents in the Department of Physiology have to undergo training in various laboratory techniques as part of the course curriculum for which they are posted in different clinical laboratories on a rotatory basis. In the present study, we evaluated the inter-observer variability between a trained technician and two academic residents posted in the andrology laboratory. This unique approach acted both as a quality control measure as well as an assessment of training outcomes.

## Materials and methods

This observational study was conducted in the Andrology Laboratory, Department of Physiology, All India Institute of Medical Sciences (AIIMS) Patna, Bihar, India. This analysis is part of a quality control program of an ongoing study that was approved by the Institutional Ethics Committee, AIIMS Patna (approval number: IEC2019372). Prior informed written consent was taken from all the individuals. Freshly obtained semen samples from 28 individuals coming to the andrology laboratory were used. Semen analysis was performed by a regular technician (T) permanently posted in the laboratory and two residents (R1, R2) on completion of their posting in the andrology laboratory. All three examined the same sample after liquefaction and proper mixing for assessment of sperm motility, sperm concentration, sperm vitality, and sperm morphology. Semen analysis was done as per the latest recommendations of the WHO [4].

Semen collection and assessment 

The participants were instructed to report after an abstinence period of two to seven days. They were provided with a wide-mouth plastic container and were instructed to avoid any contact with lubricants or soap residues. The semen samples were collected in the laboratory by masturbation. The samples were kept for liquefaction in an incubator at 37 degrees Celsius for 30 minutes. Samples with delayed liquefaction, abnormal viscosity, and insufficient volume were excluded. A manual semen examination was done.

Motility was assessed immediately after liquefaction. No dilution was done to assess motility. Each participant examined at least 200 sperms under 400X magnification in two replicate wet preparations. The mean of the two replicates was taken as the motility for that participant. Based on motility findings with appropriate dilutions (1:2,1:5,1:20,1:50) as per WHO manual guidelines, sperm count was done. All the sperms present in the center 1mmX1mm area of Improved Neubauer’s hemocytometer were counted. Sperm concentration was determined using appropriate multiplication factors according to the dilution used.

Vitality was assessed using eosin-nigrosin stain. Semen was mixed with eosin-nigrosin stain and suspension was left for 30 seconds. Smear from the suspension was prepared on a glass slide and examined under 1000X magnification. Sperm heads colored pink were counted as dead, whereas sperm heads that did not take up stain were counted as live. Uniform thickness smears were prepared and air-dried for sperm morphology assessment. They were fixed, stained, and counted under 1000X magnification based on recommended protocols. The sperms were classified as either ideal or abnormal based on observation of all parts of the sperm [4].

Statistical analysis

Data from 28 subjects was collected and entered in Microsoft Excel (Microsoft Corporation, Redmond, Washington, United States). It was analyzed using IBM SPSS Statistics for Windows, Version 22.0 (Released 2013; IBM Corp., Armonk, New York, United States). The CV for the estimates made by different participants was calculated. CV was calculated, in percentage, by dividing the standard deviation by the mean of the three participants and multiplying by 100. Intraclass correlation coefficient (ICC) estimates and their 95% confidence intervals (CI) were calculated based on a mean-rating (k = 3), absolute-agreement, 2-way random-effects model. S charts and Bland-Altman plots were made to assess the variations among the participants. Upper and lower Warnings, as well as action levels, were also determined for the S chart according to the WHO laboratory manual. Bland-Altman plots were plotted to compare two individual participants and data points outside the two standard deviations were considered out of range. Error type whether systematic or random was determined using the basic control rules for quality control charts [4].

## Results

In this study, we evaluated the interobserver variability in semen parameters on semen analysis of the same sample by three different assessors. ICC for sperm concentration, sperm vitality, sperm morphology, and sperm motility with a 95%CI was 0.982 (0.967-0.991), 0.955 (0.916-0.978), 0.490 (0.045-0.747), and 0.971 (0.945-0.986), respectively. The mean CV was computed for each semen parameter. The mean CV for sperm concentration across the samples was 6.24%. For sperm vitality, sperm morphology, and sperm motility, the mean CV was 10.14%, 2.66%, and 8.11%, respectively (Table 1).

**Table 1:** Mean of semen parameters by three observers and mean CV and range of CV among them CV: coefficient of variation

Semen Parameters	Mean	SD	Mean CV (%)	Range of CV (%)
Sperm concentration	47.80	3.03	6.24	1.2-23.02
Sperm vitality	56.78	5.75	10.14	3.68-26.24
Sperm morphology	92.24	2.45	2.66	1.05-5.75
Sperm motility	54.78	4.44	8.11	4.35-15.48

Control chart analysis

**Figure 1:**
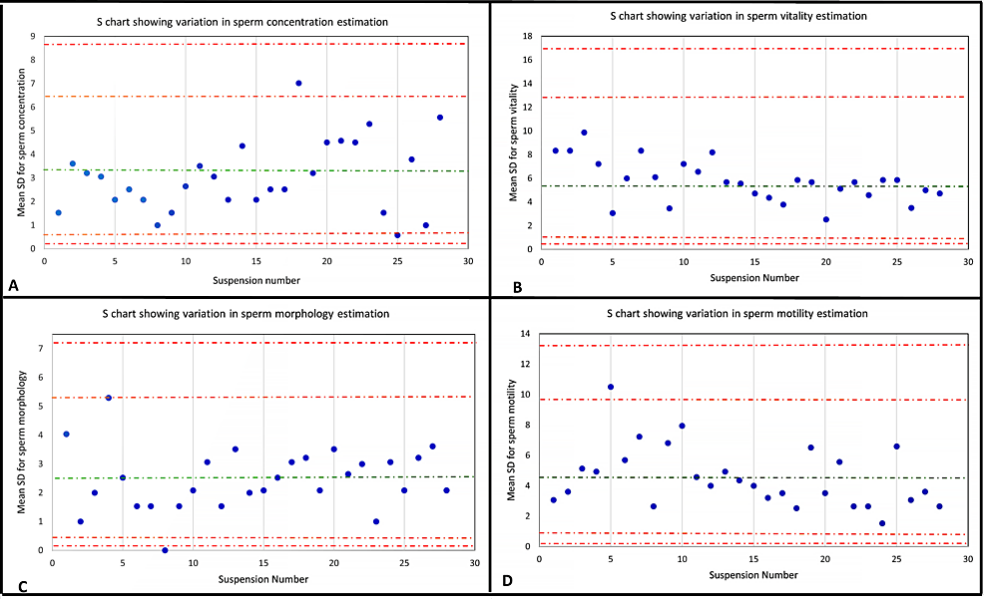
S chart showing variation in the estimation of different semen parameters (A) S chart showing variation in sperm concentration estimation; (B) S chart showing variation in sperm vitality estimation; (C) S chart showing variation in sperm morphology estimation; (D) S chart showing variation in sperm morphology estimation. Warning limits are denoted by orange dotted lines; Action limits are denoted by red dotted lines.

The S chart analysis revealed that one measurement in sperm morphology assessment fell outside the action control limits, indicating a significant deviation from the expected values. Furthermore, two measurements for sperm concentration and two for sperm vitality also exceeded the warning control limits in the S charts, suggesting potential inconsistencies in these parameters (Figure 1).

Bland-Altman plot analysis

**Figure 2:**
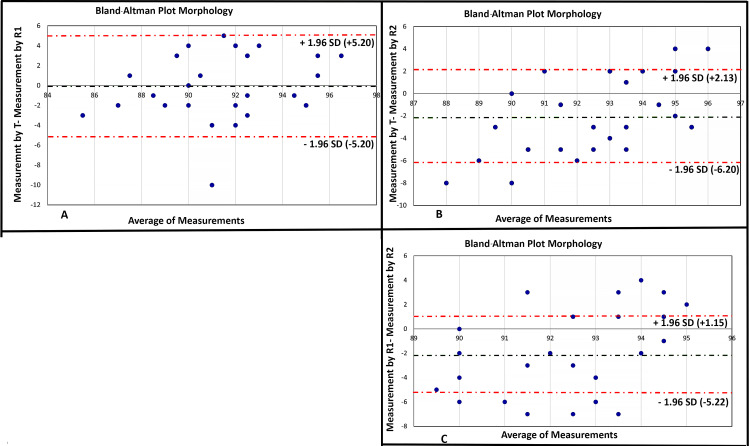
Bland-Altman plot showing interobserver variability in sperm morphology assessment (A) Bland-Altman plot between measurement of observer T minus measurement of observer R1 on y-axis and average of measurements of observers T and R1 on x-axis; (B) Bland-Altman plot between measurement of observer T minus measurement of observer R2 on y-axis and average of measurements of observers T and R2 on x-axis; (C) Bland-Altman plot between measurement of observer R1 minus measurement of observer R2 on y-axis and average of measurements of observers R1 and R2 on x-axis Red dotted lines denote ±2SD; green dotted lines denote mean of differences between measurements T: technician; R1: resident 1; R2: resident 2

In the Bland-Altman plots, variations were observed primarily in the comparisons of sperm morphology differences. Notably, the pairs T-R2 and R1-R2 exhibited values that exceeded two standard deviations of the means, indicating substantial differences in sperm morphology assessments between these pairs. However, for all other comparisons in the Bland-Altman plots, there were no significant variations detected (Figure 2).

## Discussion

Each semen sample was examined by three individuals and results were analyzed by CV, S chart, and Bland-Altman plots. The combined analysis of the Bland-Altman plots and S charts suggests the presence of a few instances of possible random errors in semen analysis. These errors were primarily observed in sperm morphology assessments, as evidenced by the Bland-Altman plots. However, it is essential to emphasize that these errors do not appear to stem from systematic issues within the semen analysis procedures. Instead, they are more likely to be sporadic deviations from the expected values, underlining the need for ongoing quality control measures and meticulous data validation in semen analysis.

There are no standard criteria for classifying the CV into groups. A lower CV indicates more agreement and therefore less variation among the observers. In some earlier studies, a CV of less than 10% was considered as good, less than 20% was considered acceptable and more than 20% was considered unacceptable [6,7].

Random errors predominantly stem from variances in measurement values or sampling processes. The complexity and subjectivity involved in semen analysis can influence the frequency at which these errors manifest necessitating a more vigilant approach to testing. Conversely, assessments employing fixed slide or video-based methodologies, characterized by a reduced number of critical procedural steps prone to errors, might require less frequent testing to maintain data quality and reliability [8].

Systematic errors, often referred to as bias, can exert a pronounced impact on research outcomes. These errors may lead to results clustering at the extremes of a data range, causing high agreement among the observers. Therefore, meticulous attention to identifying and mitigating systematic errors is imperative to maintain the integrity and accuracy of scientific findings [4,2].

Interestingly, although the mean CV percentage of sperm morphology is low, there are greater variations seen in the S-chart and Bland-Altman plots. This is not seen for other semen parameters. One reason for such results could be that, for sperm morphology, the variation in results was in a narrow range. Our results for the S chart and Bland-Altman plot are thus more sensitive for the spread of data across various semen parameters.

Cooper et al. [9] reported findings similar to the present study. The mean inter-technician CV reported by them for concentration, motility, and morphology was 6.1%, 5.6%, and 5.6%, respectively. Brazil et al. [8] studied inter-technician variability among trained technicians. They reported the mean CV for sperm count as 15.2% and for sperm motility as 10.5%. 

A few authors have argued about the relevance of quality control and quality assurance. Jequier argues that from a clinical and financial perspective, the variations in results of semen analysis do not matter, although most of the authors emphasize that quality control is important for an andrology laboratory in the investigation of male infertility. Quality control is crucial for any clinical or research outcomes in healthcare [10-17].

Despite this consensus, semen analysis laboratories across India are highly varied in terms of infrastructure, operating procedures, protocols, supervision, and reporting. Evidence for this comes from a recent survey in 2021 that reported significant differences and various inconsistencies in semen analysis methods [18]. By adhering to the WHO laboratory manual for semen analysis universally, we can establish a standardized approach to examination methods, ensuring thorough training and the implementation of crucial quality control measures. This will ultimately result in consistent reporting of semen parameters across all laboratories [4].

Limitations of the study

No comparison between the observers was made using samples having known target values. Although both known target value and unknown target value samples have their advantages and disadvantages, the use of a known target value sample could have been advantageous as it would have resulted in a more robust quality control assessment.

## Conclusions

Regular quality control assessments are essential in andrology laboratories to ensure accurate and reliable results. Proper training of laboratory personnel is also vital for consistent outcomes. While this study focused on specific quality control methods, the WHO-recommended methods are suitable for wider adoption, promoting standardized and reproducible semen analysis results. Other measures such as equipment calibration, using high-quality reagents, and standard reporting are also crucial. Further research should be aimed at assessing the impact of training on the effectiveness of quality control measures and guide the development of more robust training protocols, ultimately contributing to the overall enhancement of quality control procedures in laboratory settings.
